# Sleeping Safely! A Quality Improvement Project to Minimize Nighttime Interruptions without Compromising Patient Care

**DOI:** 10.1097/pq9.0000000000000404

**Published:** 2021-05-05

**Authors:** Clifton C. Lee, Nastassia M. Savage, Emily K. Wilson, Jennifer Brigle, Daniel Poliakoff, Rozana Shah, Tracy Lowerre

**Affiliations:** From the * Children’s Hospital of Richmond at Virginia Commonwealth University, Richmond, Va.; † Virginia Commonwealth University School of Medicine, Richmond, Va.; ‡ Mainstreet Pediatrics, Towson, Md.; § Children’s Specialty Group, Norfolk, Va.

## Abstract

Sleep is crucial for patients’ health but is often disrupted, slowing recovery and resulting in adverse health effects. This study identified whether passive vital sign checks (heart rate, respiratory rate, and pulse oximetry) and delayed routine morning laboratories in clinically stable pediatric patients minimized nighttime interruptions without compromising patient safety.

**Methods:**

After developing the inclusion criteria using the Pediatric Early Warning Score, we enrolled eligible patients for the intervention. We assessed physician compliance through order entry and nursing compliance through recorded vital signs and timing of blood draws. Eligible patients received passive vital sign checks at 4 am with routine morning laboratories drawn at midnight or 6 am, instead of 4 am, to minimize patients’ nighttime interruptions. All other nursing duties continued with the institution’s patient care policies. Finally, retrospective chart reviews were performed to determine whether the intervention resulted in the escalation of care, our primary outcome.

**Results:**

We collected 2,138 individual data points, which represented approximately 420 patients. Over the intervention period, high compliance rates with physician order placement, nurse performing passive vital signs, and delayed blood draws were maintained. On eligible patients, there was no escalation of care or rapid response team involvement.

**Conclusions:**

The use of passive vital sign checks on eligible pediatric patients was generally well-received and had high compliance during the intervention period. There were no negative patient care consequences, supporting the feasibility of this program. Further studies are needed to determine sleep quality and patient satisfaction.

## INTRODUCTION

Sleep is crucial for both physical and mental health and has therapeutic capabilities. Despite this, sleep is often overlooked in hospitalized patients, although many biological processes are modulated by sleep cycles.^1^ Poor sleep has been linked to numerous adverse health effects, including increased stress hormone levels, altered immune function, cognitive dysfunction,^2,3^ significantly higher levels of pain,^4^ obesity,^5^ and impaired healing.^6^ Also, sleep interruption in hospitalized patients is a significant source of stress and anxiety.^7^ Furthermore, pediatric patients are more tired when they are awoken more frequently at night^8^ with 1 study reporting nearly 60% of patients being sleepy during the day and 52% needing a nap.^9^

There are various reasons that pediatric patients may have their sleep disturbed, including noises such as alarms, machines, pagers, talking, footsteps, and people entering and exiting the room.^8,9^ However, more disruptive than the noises were collecting vital signs and assessments, with some patients experiencing these between 6 and 10 times a night; specifically, 31% of patients have laboratory draws, 85% receive medications, 48% are fed, and 62% have IV lines checked.^9^ By minimizing some of these interruptions, it is possible that patients should be able to get better rest and shorten the duration of hospitalization, with a lower likelihood of complications.^10,11^

Many hospitals currently assess vital signs (ie, heart rate, respiratory rate, pulse oximetry measurements, temperature, and blood pressure) at regular intervals every four hours and collect routine blood draws for laboratory testing in early morning hours, regardless of clinical indication. Although these traditional practices remain prominent, there is limited evidence to suggest that it is beneficial. Yoder et al^12^ determined that 45% of adult patients with a Medical Early Warning Score of ≤1 were still awakened overnight for vital sign checks, despite a less than 1% chance of an adverse event. This finding suggests that it would be safe and more cost-effective to minimize distributing patients’ sleep, as the current practice, when it has few benefits to the quality of care.

At the study institution, an urban, academic university medical center, it is the norm to perform vital signs every 4 hours on all hospitalized, non-PICU patients and obtain routine laboratory studies in the early morning hours, usually at 4 am. As a result, the physician and nursing staff collaborated to create a quality improvement (QI) initiative to minimize nighttime interruptions in clinically stable hospitalized pediatric patients by utilizing passive, noninterruptive vital signs (ie, heart rate, respiratory rate, pulse oximetry measurements) overnight and changing the time of routine laboratory draws.

This QI initiative’s primary goal was to assess whether minimizing nighttime interruptions would negatively impact patient safety as measured by an increase in transfers to the Pediatric Intensive Care Unit (PICU) or rapid response team (RRT) activations. We determined the balancing measure, as the number of unplanned or unexpected patient transfers to the PICU and RRT activation on eligible patients. The global SMART aim of this initiative was to employ passive vital sign checks and delayed routine morning labs in eligible patients with 80% compliance in physician order entry, 90% compliance in passive vital sign usage, and 90% compliance in routine lab draw time change achieved within a year of implementation.

## METHODS

We formed an interdisciplinary group for the QI initiative, initially comprised a pediatric hospitalist, 2 pediatric residents, 2 pediatric nurses, and a nurse clinician. A parent from the Children’s Hospital of Richmond Family Advisory Network was later added to the team who provided feedback regarding the intervention process. The Children’s Hospital of Richmond at Virginia Commonwealth University is an urban academic university medical center with a 49-bed acute care pediatric (ACP) unit and a 20-bed PICU. This group intended to identify ways to minimize nighttime interruptions in a subset of patients admitted to the ACP unit who were considered clinically stable without compromising their safety. The following sections describe the participants for this effort, the data collection, and the measures used.

### Patient Population and Baseline Data

From June 21 to July 18, 2016, baseline data were collected. We reasoned that the 1-month time period was a fair representation of the breath of hospitalized patients. From this review of the baseline data, the QI team consisting of physicians and nurses developed the inclusion and exclusion criteria. Specifically, eligible patients were those who met all of the following inclusion criteria: age 10 years and older, medical (nonsurgical) patients admitted to the ACP unit, not receiving opiate medication in the last 12 hours, not requiring frequent vital sign checks for medications or infusions (ie, intravenous immunoglobulin or packed red blood cell transfusion), Pediatric Early Warning Score (PEWS) ≤2 at 8 pm and midnight vitals, and physician and parents/guardians had not requested a full set of vitals at 4 am. We chose the age of 10 years because older children were likely to answer nurses’ questions regarding pain without parental help as some patients did not have parents staying with them. The total PEWS of 2 or less was chosen because no active intervention was needed based on the institution’s PEWS algorithm. Patients admitted to the intermediate care unit, or PICU would need frequent monitoring by physicians and nurses. Surgical patients were excluded because the surgical services did not wish to participate in the QI initiative. Finally, the rest of the exclusion criteria would require frequent nursing assessments. Using these criteria, we identified 166 patient encounters within this time frame. Through patient chart review, none of these patient encounters had an RRT activation or a transfer to a higher level of care such as the PICU. They did not benefit from the acquisition of overnight vital signs.

During the QI initiative, the admitting physician reviewed patients who met the inclusion criteria to determine whether passive vital sign checks were appropriate. If so, the physician would enter a Clinical Communication order on the electronic medical records (EMRs). The order instructed the nurse to perform passive vital signs check at 4 am and obtain blood for routine laboratory testing at either 12 midnight or 6 am. Patients who met the exclusion criteria received standard vital sign checks every 4 hours, and blood was drawn at 4 am for routine laboratory studies. Passive vitals included monitoring and recording heart rate, respiratory rate, and pulse oximetry measurements as they can be obtained without waking the patient. Temperature and blood pressure measurements were withheld as these often wake patients or require their participation to collect them.

### Measures

**Fig. 1.**
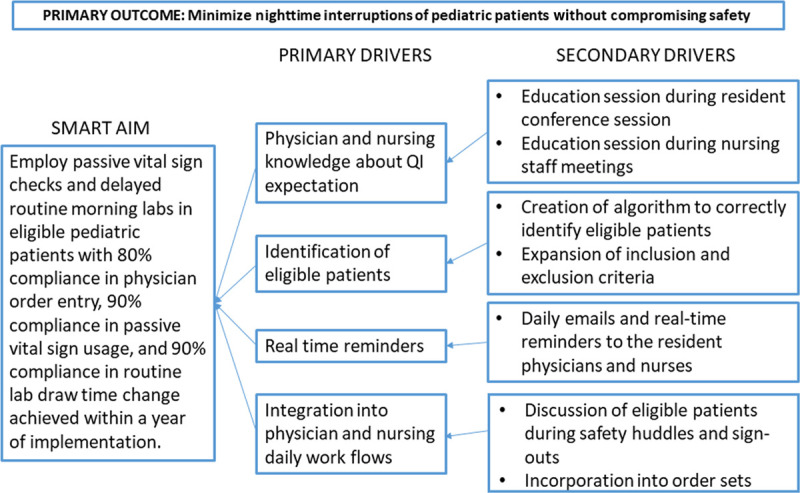
Key driver diagram.

As shown in the key driver diagram (Fig. 1), the following were collected, assessed, and monitored during the QI initiatives duration: the number of eligible patients; whether a provider placed a clinical communication order; type of vitals (ie, full set vs. passive) recorded; PEWS at 8 pm and 12 midnight to ensure patients remained stable; and the timing of routine laboratory blood draws. Within a year of implementation, we aimed to achieve 80% compliance on order entry, 90% compliance on the appropriate timing of routine laboratory blood draws, and 90% compliance on the type of vital signs collected at 4 am. The primary outcome was to determine the patient safety of minimizing nighttime interruptions. We determined this from the balancing measure, the number of patient transfers to the PICU and/or RTT activation on eligible patients during the QI implementation period. A member of the QI team accessed the EMR of eligible patients daily to collect the necessary data.

### Interventions

Before the QI initiative, we planned educational sessions for residents and nursing teams. We announced the QI project during resident morning conference and nursing staff meetings. Emails describing the QI initiatives and the inclusion and exclusion criteria were sent to the pediatric faculty, residents, and nursing leadership on the ACP unit. Despite the planned announcements, physician order entry compliance and nursing compliance in selecting passive vital sign checks for eligible patients were below goal percentiles. During the first PDSA cycle beginning in week 6, the QI team sent daily informational reminder emails as a feedback mechanism to attendings and residents to improve the process. Simultaneously, the nurse clinicians provided educational sessions to the nursing staff on using the clinical communication order, determining whether patients met eligibility criteria for passive vitals, and appropriate rescheduling of routine laboratory blood draws. Also, for ease of understanding and to create a visual reminder, the QI team created an algorithm for patient eligibility selection and shared it with the resident and nursing teams. We posted this algorithm in the nurses’ stations and resident workrooms on ACP units. Finally, nurses were encouraged to ask physicians for the clinical communication order on eligible patients.

During the second PDSA cycle beginning in week 13, we expanded the exclusion criteria to include patients who required neurologic checks every 4 hours or had scheduled Albuterol treatments. We excluded these patients at the nurses’ request because they required a nursing intervention that awakened the patients every 4 hours. The resident and nursing teams continued to receive daily reminder emails regarding eligible patients and process measure goals.

During the third PDSA cycle beginning in week 37, nursing discussion regarding the use of passive vital signs on eligible patients during nightly safety huddles started. Reminder emails continued to be sent to residents and nurses to encourage passive vital signs during patient sign out. Finally, nurses were empowered to discuss eligible patients with the residents. These interventions were also supported by feedback requesting improved communication among the resident and nursing teams. From weeks 45 to 52, data were not collected daily; therefore, the QI team decided not to include the data from those weeks on the control chart.

During the fourth PDSA cycle beginning in the second week of the second year, we altered the inclusion criteria to include children 5 years of age or older. This decision was made after analyzing the data from eligible patients and concluding that no eligible patients required PICU transfers or RRT activation. Additionally, the pediatric hematology/oncology admission order set in the EMR included a clinical order to perform passive vital signs on eligible patients.

Finally, during the fifth PDSA cycle beginning in the eighth week of the second year, we included the clinical order for passive vital signs in the EMR admission order set for all pediatric medical patients.

### Statistical Analysis

We used statistical process control with our process measures of physician adherence to passive vital signs order entry, nursing adherence to appropriate vital signs performed, and nursing adherence to changing the timing of routine laboratory blood draws. Data were displayed on a u-chart. We used established rules for differentiating special versus common cause variation for this chart. Regarding the patient safety aspect of performing the QI initiative, no statistical analysis was performed as no unexpected PICU transfers or RRT activation occurred.

### Ethical Considerations

We obtained local Institutional Review Board approval for the QI initiative. All members of the QI team completed the Collaborative Institutional Training Initiative Program before the start of the QI initiative.

## RESULTS

The QI initiative began on September 7, 2016, and the QI team conducted daily chart reviews of the eligible patients. The final analysis included 2,138 data points; each represented 1 day for each day the eligible patients were hospitalized. Some eligible patients with a longer length of stay had more data points since we analyzed charts daily. Therefore, we have extrapolated the approximate number of eligible patients to be 420 based on the average length of stay of 5 days on the ACP unit during the study period. We constructed the run charts and updated data weekly to map the progress of the process measure goals.

**Fig. 2.**
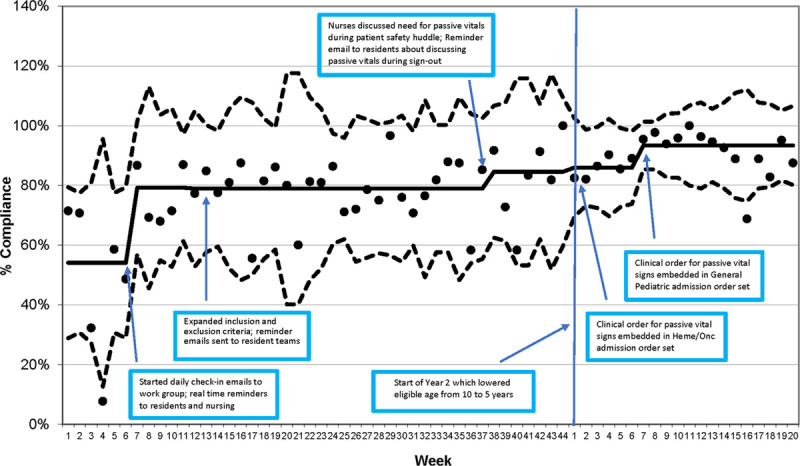
Clinical order entry compliance rates.

Before the first PDSA cycle, the physician order placement compliance rate was below the 80% goal rate. The potential causes of this change were discussed, and inpatient residents’ turnover every 2 weeks was determined to be the primary cause. To improve the compliance rates, the QI team sent daily emails and real-time reminders to medical and nursing teams to consider whether each patient was eligible for QI project enrollment. These efforts resulted in an improved compliance rate of a median of 79.3%, which was just below the 80% compliance goal. After focusing on improving nursing and resident physician communication, the compliance rate improved to a median of 85%. Order placement compliance was not significantly impacted by expanding the inclusion age from 10 to 5 years of age and older. However, adding an electronic order set for general pediatrics admissions increased the median compliance rates to 93% (Fig. 2).

**Fig. 3.**
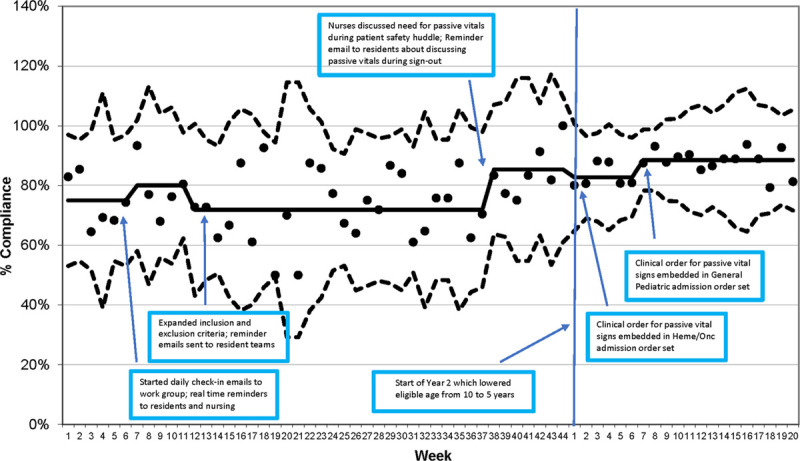
Vitals at 4 am compliance rates.

During the first 2 PDSA cycles, the compliance rate for appropriate vital signs completed at 4 am was below the goal rate, with a median between 70% and 80%. After the focus on improving communication, there was an increased median compliance rate of 85%. Expanding the age of inclusion from 10 to 5 years of age and older did not significantly impact the compliance rate. The compliance rate improved to a median of 89% after adding an electronic order set (Fig. 3).

**Fig. 4.**
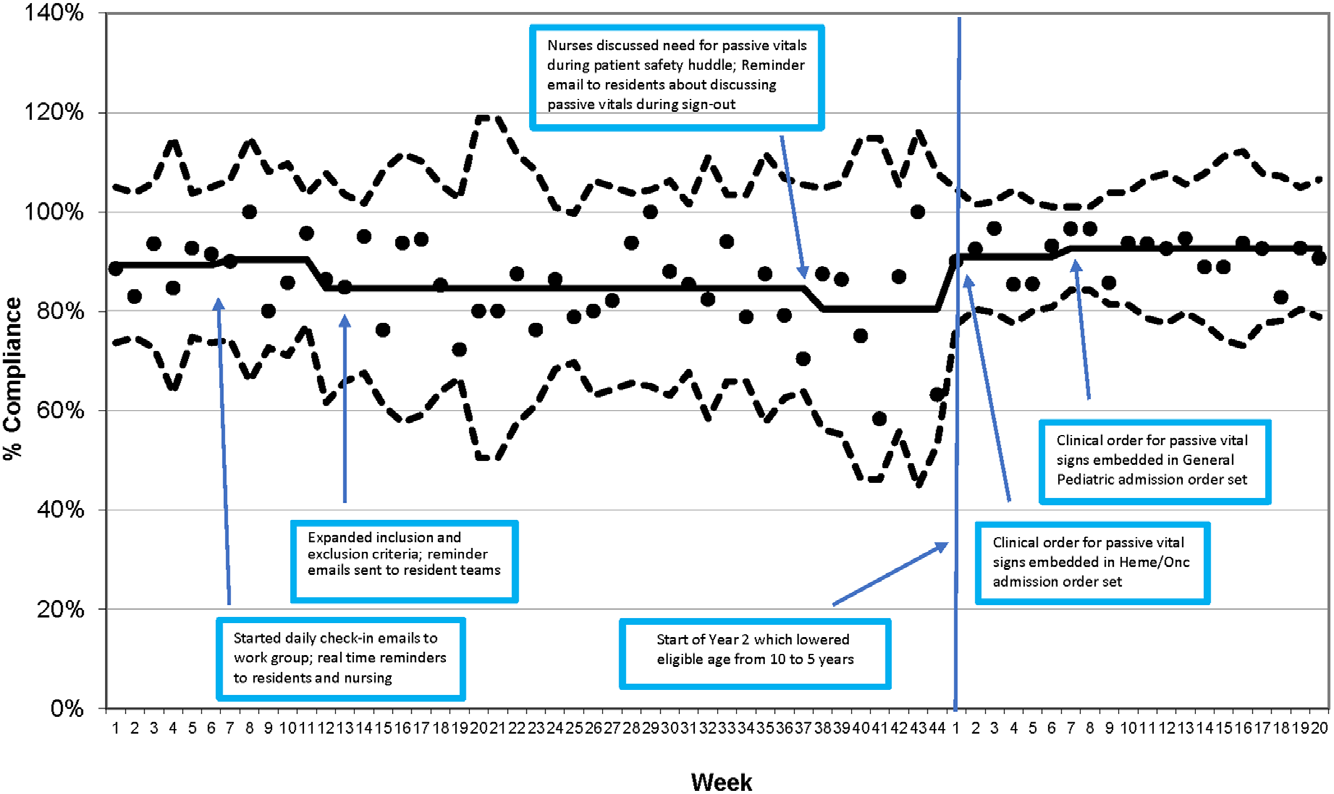
Appropriate laboratory compliance rates.

Compliance rates for changing the timing of routine laboratory blood draws for eligible patients were at goal with a median of 89%–90%; however, this decreased to 80%–85% during most of the QI initiative. We surmise that nurses were very excited at the prospect of delaying blood draws for routine laboratory studies for eligible patients, thus explaining the high compliance rate. The compliance rate did drop slightly; however, the overall compliance rate for routine laboratory blood draw times remained high. Expanding the age of inclusion from 10 to 5 years of age and older increased the median to 91%–93%, where it remained for the remainder of the QI project data collection (Fig. 4). During this QI initiative, we continuously reviewed the EMR of eligible patients to ensure that passive vital sign checks did not negatively impact the quality of care. None of the eligible patients required escalation of care defined as RRT activation or unexpected transfers to the PICU.

## DISCUSSION

It is well known that sleep aids in a patient’s recovery. It is also known that a variety of factors affect sleep quality.^13^ Delaney et al^14^ conducted a cross-sectional study involving 15 clinical units within a 672-bed tertiary referral hospital in Australia. The study concluded that hospitalized patients are exposed to factors that reduce the duration and quality of sleep. The findings from this study provided the foundation for a QI project. Like Delaney’s study, our QI initiative stem from the premise that hospitalized patients have diminished duration and quality of sleep. However, our QI initiative supports the premise that minimizing nighttime interruptions in clinically stable hospitalized pediatric patients is possible and beneficial. By initiating passive vital signs and changing the timing of routine laboratory blood draws, this effort identified a more patient-centered option that appears to be a safe alternative for monitoring and requires fewer interruptions. Multiple PDSA cycles incorporating feedback through safety huddles, emails, and reminders to residents and nursing achieved substantially improved compliance rates. It also maintained high participation levels from physician and nursing staff in the use of passive vital sign checks and routine laboratory blood draws rescheduling. Overall, using passive vital sign checks and alternative timing for routine laboratory blood draws did not have a negative impact. It provided new insight into 1 method for reducing nighttime interruptions. We are unaware of other QI initiatives that minimize nighttime interruptions and assess patient safety in hospitalized pediatric patients.

## LIMITATIONS

This QI initiative has limitations to its generalizability, including assessing process and balancing measures in 1 pediatric inpatient unit at a single institution. It would benefit from replication in other institutions. Second, we assessed the initiative’s impact on compliance rates but not the quality and quantity of the patients’ sleep and patient satisfaction. There are many external and uncontrollable factors, such as having a roommate, the beeping of monitors, and scheduled care. Third, there is a possibility that even passive vital signs could have woken up the patient. We did not account for this possibility by gathering data from nurses about the 4 am vital check. Fourth, this initiative was limited to nonsurgical patients; future research should consider the potential utility of passive vital sign checks for this population. Fifth, eligible patients remained on continuous monitoring to gather passive vital signs of heart rate, respiratory rate, and pulse oximetry. Monitor alarms may have awakened the patient. Also, continuous pulse oximetry goes against Quinonez et al’s *Choosing Wisely* in pediatric hospital medicine’s recommendation.^15^ Additional efforts to minimize nighttime interruptions should avoid continuous monitoring for eligible patients. Finally, although we did expand the eligible patient age to 5 years and older, we have no data on younger children. Future initiatives could expand the eligible age even further to include all but infants who still require frequent feedings overnight.

## CONCLUSIONS

Sleep is an essential factor in the overall healing process. Traditional care practices such as around the clock vital sign checks involve sleep interruptions, which are counterproductive to the overall healing process. This QI project tested whether passive vital sign checks and alternative times for blood draws for clinically stable pediatric patients had any adverse patient outcomes. This study demonstrated that a high level of care delivery could be safely maintained by minimizing nighttime interruptions. This approach should allow patients to get more rest and improve their recovery processes. Future research should assess the impact of passive vital signs and delayed laboratory blood draws on the quality and quantity of sleep and patient satisfaction.

## DISCLOSURE

The authors have no financial interest to declare in relation to the content of this article.
